# Supplementary figures and images for: Single‐cell analysis identified lung progenitor cells in COVID‐19 patients
Source: Cell Prolif. 2020 Oct 22;53(12):e12931. doi: 10.1111/cpr.12931 (PMC7645905; doi:10.1111/cpr.12931)

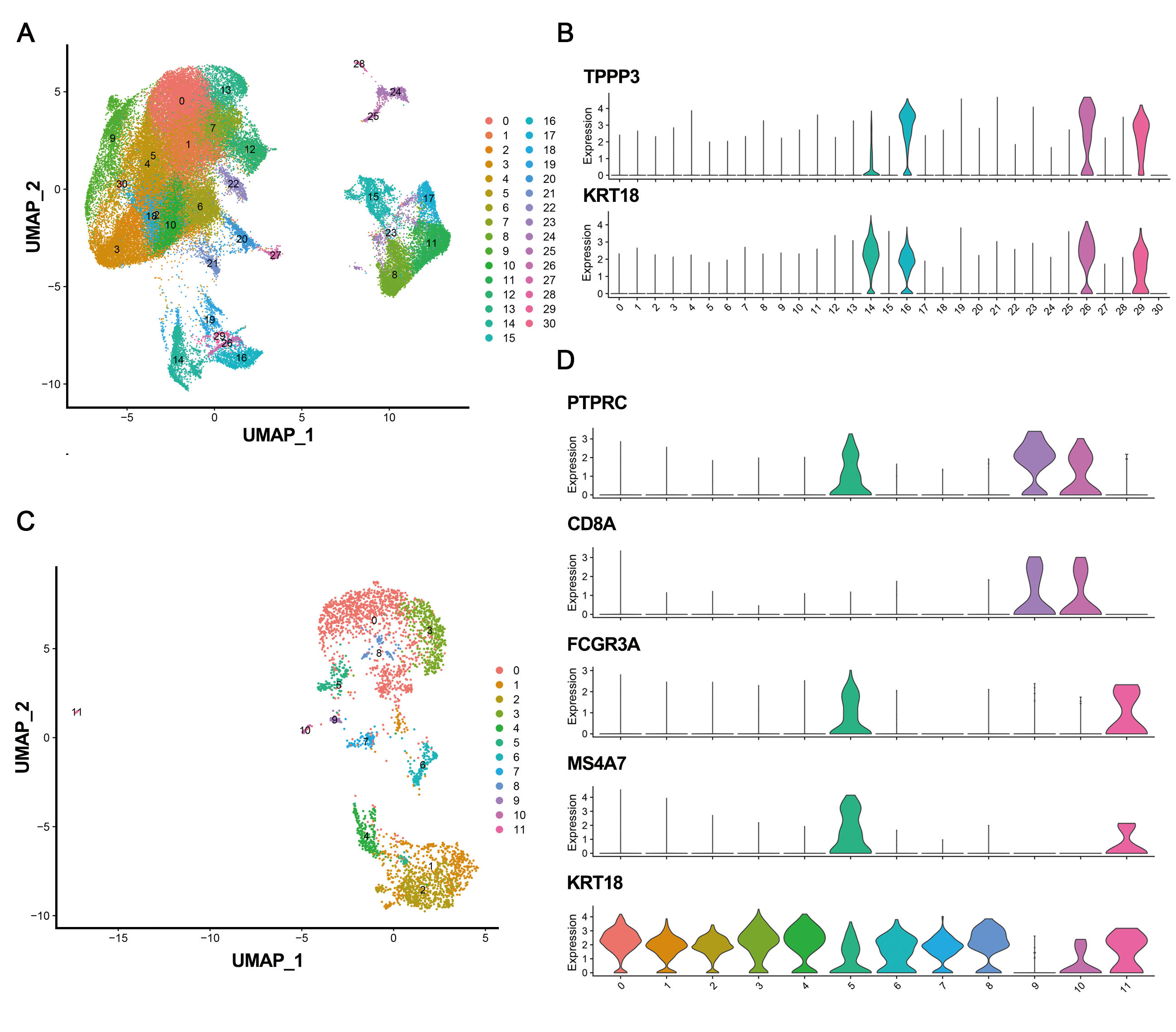

Supplement: Supplementary file 1 — Figure S1 [file CPR-53-e12931-s001.png]

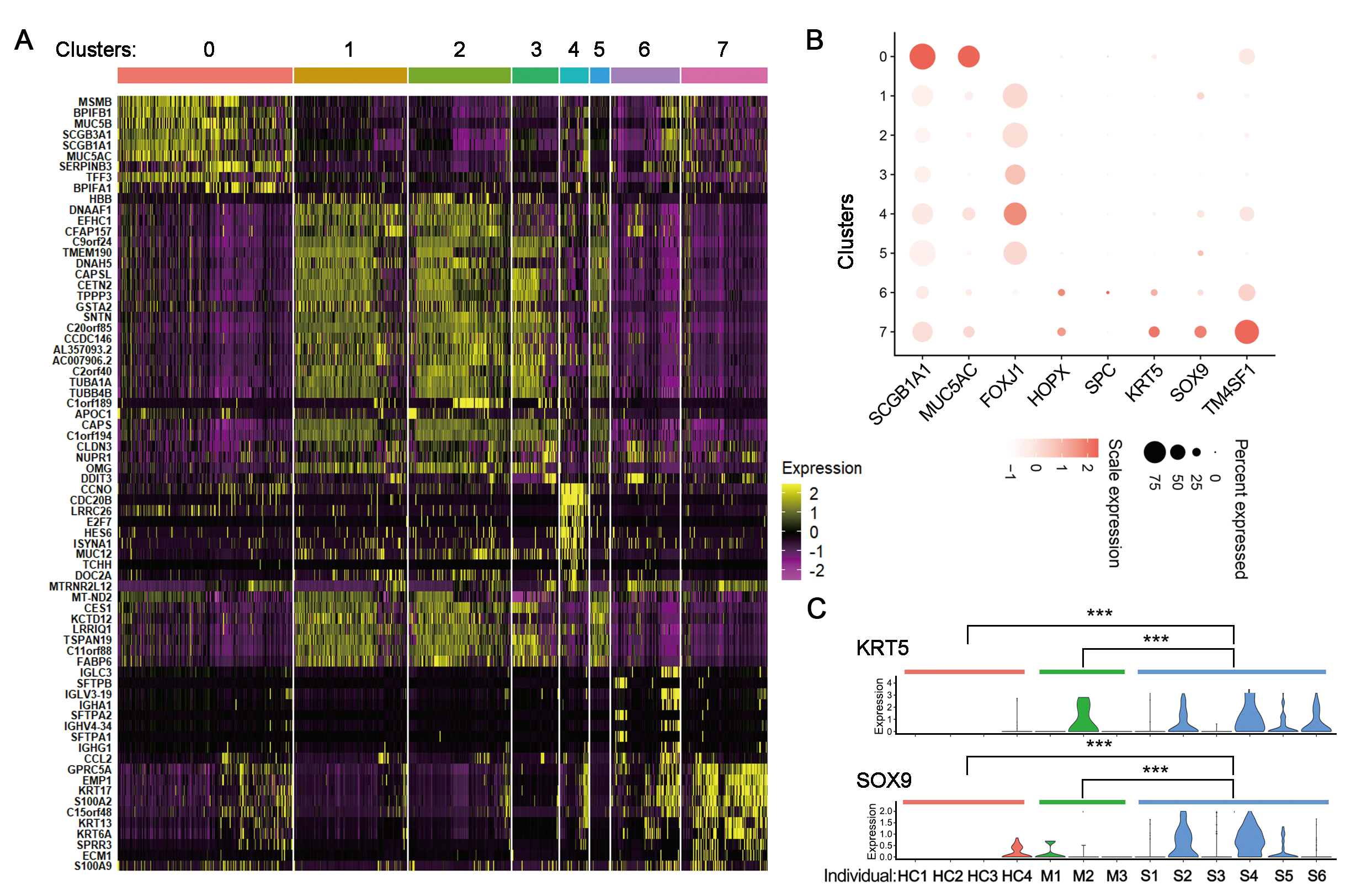

Supplement: Supplementary file 2 — Figure S2 [file CPR-53-e12931-s002.png]

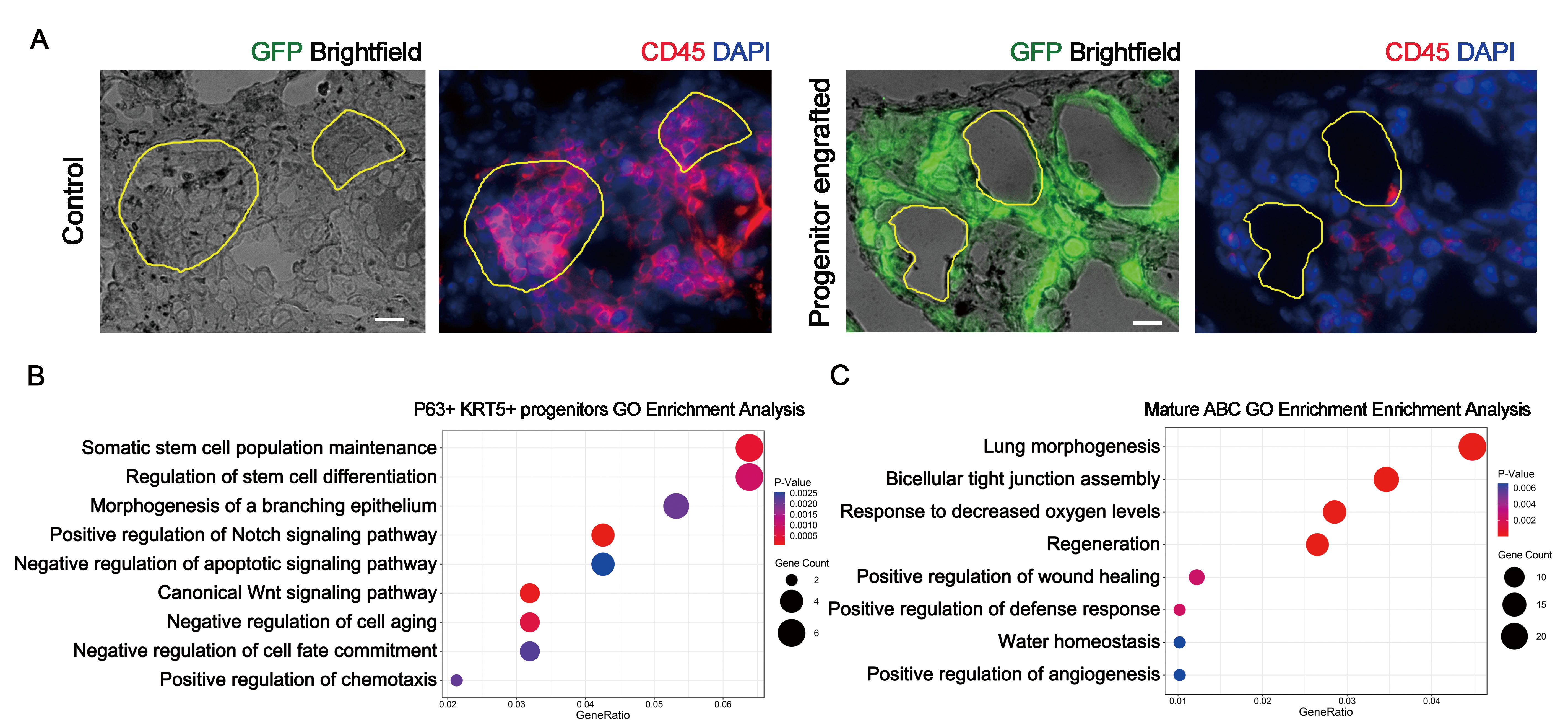

Supplement: Supplementary file 3 — Figure S3 [file CPR-53-e12931-s003.png]
