# Supplementary material for: Single‐cell analysis identified lung progenitor cells in COVID‐19 patients
Source: Cell Prolif. 2020 Oct 22;53(12):e12931. doi: 10.1111/cpr.12931 (PMC7645905; doi:10.1111/cpr.12931)
Supplement: Supplementary file 4 — Supplementary Material [file CPR-53-e12931-s004.docx]

**Figure S1**

ScRNA-seq analysis of all BALF cells. A, UMAP presentation of all major cell types in BALF (n = 13). B, The gene expression levels of epithelial cell markers in each cluster (n = 13). C, The original UMAP presentation of major cell types in BALF epithelial cells without removing leucocyte-engulfed cells. (n = 13). D, The gene expression levels of immune cell markers in original BALF epithelial cells. Cluster 5 (FCGR3A+ MS4A7+), 9 (PTPRC+ CD8A+), 10 (PTPRC+ CD8A+) and 11 (FCGR3A+ MS4A7+) were excluded in further analysis (n = 13).

**Figure S2**

Identification of clusters from BALF epithelial cells. A, Heat map of the top ranked genes highly expressed in each cluster. Colour scheme is based on z-score distribution from -2 (purple) to 2 (yellow). B, A dot plot showing expression of hallmark genes by different cell types in BALF epithelial cells. (n = 13). C, The gene expression levels of KRT5 and SOX9 in cluster 7 lung progenitors from healthy controls (n = 4), moderate cases (n = 3) and severe cases (n = 6). ***P < 0.001.

**Figure S3**

Transplanted KRT5+ progenitors differentiate into alveolar barrier cells. A, Massive immune cell infiltration into alveolar cavity in non-transplanted control lung but not in transplanted lung with GFP+ progenitor engrafted. Scale bar, 25 μm. B and C, Gene Ontology enrichment analysis of the differentially expressed genes identified in P63+ KRT5+ progenitors and mature alveolar barrier cells.
